# Targeting Human Thrombus by Liposomes Modified with Anti-Fibrin Protein Binders

**DOI:** 10.3390/pharmaceutics11120642

**Published:** 2019-12-02

**Authors:** Hana Petroková, Josef Mašek, Milan Kuchař, Andrea Vítečková Wünschová, Jana Štikarová, Eliška Bartheldyová, Pavel Kulich, František Hubatka, Jan Kotouček, Pavlína Turánek Knotigová, Eva Vohlídalová, Renata Héžová, Eliška Mašková, Stuart Macaulay, Jan Evangelista Dyr, Milan Raška, Robert Mikulík, Petr Malý, Jaroslav Turánek

**Affiliations:** 1 Laboratory of Ligand Engineering, Institute of Biotechnology, Czech Academy of Sciences, v.v.i., BIOCEV Research Center, Průmyslová 595, 252 50 Vestec, Czech Republic; hana.petrokova@ibt.cas.cz (H.P.); milan.kuchar@ibt.cas.cz (M.K.); 2 Department of Pharmacology and Immunotherapy, Veterinary Research Institute, v.v.i., Hudcova 70, 621 00 Brno, Czech Republic; masek@vri.cz (J.M.); viteckova@vri.cz (A.V.W.); bartheldyova@vri.cz (E.B.); kulich@vri.cz (P.K.); hubatka@vri.cz (F.H.); kotoucek@vri.cz (J.K.); knotigova@vri.cz (P.T.K.); vohlidalova@vri.cz (E.V.); hezova@vri.cz (R.H.); maskova@vri.cz (E.M.);; 3 Department of Biochemistry, Institute of Hematology and Blood Transfusion, U nemocnice 2094/1, 128 20 Praha 2, Czech Republic; Jana.Stikarova@uhkt.cz (J.Š.); Jan.Dyr@uhkt.cz (J.E.D.); 4 Malvern Instruments Ltd., Enigma Business Park, Grove Lane, Malvern WR14 1XZ, UK; Stuart.Macaulay@malvern.com; 5 Department of Immunology, Faculty of Medicine and Dentistry, Palacky University Olomouc, Hněvotínská 3, 775 15 Olomouc, Czech Republic; 6 The International Clinical Research Center ICRC and Neurology Department of St. Anne’s University Hospital in Brno, Pekařská 53, 656 91 Brno, Czech Republic; robert.mikulik@fnusa.cz

**Keywords:** fibrin, thrombus targeting, thrombus imaging, binding protein, ABD scaffold, liposome, combinatorial library, metallochelation, fibrinogen Bβ chain

## Abstract

Development of tools for direct thrombus imaging represents a key step for diagnosis and treatment of stroke. Nanoliposomal carriers of contrast agents and thrombolytics can be functionalized to target blood thrombi by small protein binders with selectivity for fibrin domains uniquely formed on insoluble fibrin. We employed a highly complex combinatorial library derived from scaffold of 46 amino acid albumin-binding domain (ABD) of streptococcal protein G, and ribosome display, to identify variants recognizing fibrin cloth in human thrombus. We constructed a recombinant target as a stretch of three identical fibrin fragments of 16 amino acid peptide of the Bβ chain fused to TolA protein. Ribosome display selection followed by large-scale Enzyme-Linked ImmunoSorbent Assay (ELISA) screening provided four protein variants preferentially binding to insoluble form of human fibrin. The most specific binder variant D7 was further modified by C-terminal FLAG/His-Tag or double His-tag for the attachment onto the surface of nanoliposomes via metallochelating bond. D7-His-nanoliposomes were tested using in vitro flow model of coronary artery and their binding to fibrin fibers was demonstrated by confocal and electron microscopy. Thus, we present here the concept of fibrin-targeted binders as a platform for functionalization of nanoliposomes in the development of advanced imaging tools and future theranostics.

## 1. Introduction

Thrombosis, a critical event consisting of formation of thrombus in blood vessels, is one of the most frequent causes of death, e.g., due to ischemic stroke [1], myocardial infarction [2] or pulmonary embolism [3]. Such diseases are not only leading causes of death but have huge socioeconomic impacts worldwide despite available therapy [4]. There is clearly a significant medical need for better and rapid diagnosis and targeted treatment of acute thrombosis.

Direct thrombus imaging can be mediated by targeting surface structures of activated platelets, for instance, by single-chain antibody conjugated iron oxide microparticles [5,6]. Activated platelets can be targeted also by cyclic RGD peptides that were designed, synthesized and tested as ligands for highly specific targeting mediated by theranostic nanoparticles [7]. Another compound of the blood thrombus that can be used for targeting of the imaging agents is fibrin in the form of insoluble net of fibers. As fibrin is the end-product of proteolytic cleavage of fibrinogen followed by further coagulation process, it shares substantial sequence identity (98%) and structural similarity to its parental soluble progenitor. Concentration of fibrinogen in plasma is dominating (2–4 mg/mL), therefore, a high selectivity of fibrin-targeting ligands is required to avoid their complete scavenging by fibrinogen during in vivo circulation. To overcome this burden, several laboratories tried to develop monoclonal antibodies specific to fibrin but those recognize soluble fibrin and its D-dimer as well, or substantially cross-react with fibrinogen [8,9,10,11].

Beside antibodies, several small peptide-based ligands specifically binding fibrin were also generated. The small cyclic peptides Tn6, Tn7 and Tn10 specifically bind to fibrin and fibrin-degradation products with micromolar affinity, whereas binding to fibrinogen is about 100-fold weaker [12]. The modified version of Tn6 peptide, the peptide EP-2104R [13], was tested for thrombus imaging by MRI in the phase II clinical trials [14] but did not advanced to the next phase. In another attempt, the cyclic peptides CLT1 and CLT2 were developed to recognize fibrin-fibronectin complexes in plasma thrombi in tumors and at injured tissues sites [15].

Generation of insoluble fibrin-specific agents represents a significant challenge in the development of thrombus-specific in vivo diagnostic probes. Recently developed monoclonal antibody (mAb) clone 102-10 [16] distinguishes fibrin thrombi from precursors such as fibrinogen, soluble fibrin as an early polymerization product and insoluble fibrin precursor and degradation clot product such as D-dimer. Detail analysis of the binding specificity of this mAb identified a prominent hydrophobic region of 16 amino acid located on the Bβ chain as an exclusive epitope for binding of 102-10 mAb to fibrin thrombi [16]. This Bβ peptide epitope has been postulated to be shielded by a steric hindrance in the soluble fibrinogen but exposed in polymerized insoluble fibrin fibers. In addition, radiolabeled 102-10 mAb selectively accumulated in mouse spontaneous tumors and identified increased fibrin deposition in grade 4 glioma in comparison to lower-grade gliomas [16,17,18]. Tissue plasminogen activator (tPA) (an endogenous protein that has been shown to bind fibrin with high affinity) was studied as possible targeting ligand which might circumvent antibody difficulties. The use of tPA-derived proteins however, requires neutralization of remove the plasminogen-activating proteolytic activity. Targeting of echogenic liposomes towards fibrin was demonstrated, but no clinical application has not been demonstrated up to present [19,20].

Phage display technology was also used for identification of fibrin-specific antibodies or their fragments. Human single-chain antibody fragment Tomlinson I and J libraries were panned against non-cross-linked fibrin [21] and only one clone (E4) was identified that, in ELISA, showed a weak preferential binding to fibrin in comparison to fibrinogen. Monoclonal antibody AP2 was selected by phage display from a combinatorial library targeted to N-terminal peptide of α-chain of fibrin [22]. This antibody recognizes five N-terminal amino acids from fibrin and does not react with fibrinogen. The AP2 antibody also inhibits fibrin thrombus formation and localizes in fibrin-rich tumors as shown in vivo on a mouse model. All above mentioned antibodies, however, were not used for development of methods for direct thrombus imaging.

Various functionalized nanoparticles have already been tested for the purpose of in vivo imaging, but most of the developed nanoparticle types have not been yet approved for clinical applications. Liposomes, self-assembled membrane-like spherical vesicles, are non-toxic biocompatible nanoparticles approved by FDA and EMA for the application in human medicine with a considerable potential as diagnostic and theranostic carriers applicable for improving many imaging techniques such as Computed Tomography (CT) or magnetic resonance imaging (MRI) [23]. Several different liposomal preparations are in use as vaccines or for the treatment of infectious diseases, cancer or dermatological disorders [24,25,26]. Technologies for preparation and production of liposomes at industrial scale are available and bioconjugate chemistry for surface modifications of liposome by ligands of various chemical structure are currently being developed [27,28,29].

Therefore, we use the new approach based on ribosome display technology to identify fibrin-specific artificial protein binders derived from a small protein domain scaffold. This approach represents a valuable alternative for production of robust and high-affinity binders with a required selectivity. In this study, we used a highly complex combinatorial library derived from scaffold of albumin-binding domain (ABD) of streptococcal protein G [30,31,32], and ribosome display, to identify fibrin-specific protein binders that could be used as components for targeted delivery of nanoliposomes to human thrombi in vitro and *in vivo*. We demonstrate that one of the selected candidates preferentially binds to insoluble fibrin as well as to human thrombus *in vitro*. Thus, this ABD-derived variant can serve as a useful molecular tool for the functionalization of a liposomal surface. The concept was proved in vitro by application of flexible silicone replica of coronary artery as a model for visualization of thrombi by fluorescently labeled liposome-binder complexes under flow conditions. Nanoliposomes modified by small protein binders represent a basis for development of platform for MRI or CT imaging of thrombus as well as for targeted delivery of thrombolytic drugs or theranostics when combined together.

## 2. Materials and Methods

### 2.1. Production of Fusion Proteins Carrying Bβ Epitopes (BEP) Recognized by 102-10 mAb

For the construction of triple-Bβ-containing protein target (3BEP-TolA-Avi), the codon-optimized DNA sequences of triple-Bβ epitope (CNIPVVSGKECEEIIR) connected with GGGGS hinges was synthetized by GeneArt (Regensburg, Germany) and inserted into pET28b vector between N-terminal His-tag and C-terminal TolA-Avi-tag using digestion with NcoI and BamHI enzymes. Protein was expressed in *Escherichia coli (E. coli)* BL21 (DE3) *BirA* strain as in vivo biotinylated product. Purification was done using 1 mL NiNTA-agarose matrice (Qiagen, Hilden, Germany) under native conditions. Column with matrice was equilibrated with TNI20 buffer (50 mM Tris, 300 mM NaCl and 20 mM imidazole pH 8.0) and the sonicated protein culture in TNI20 buffer was applied twice and washed with 10 mL of the same buffer. Elution was done by TNI250 (50 mM Tris, 300 mM NaCl and 250 mM imidazole, pH 8.0). Fractions with highest concentration of protein were pooled and polished by size exclusion chromatography on Superdex 200 10/300 column in the TN buffer (50 mM Tris, 150 mM NaCl, pH 8.0).

Fusion protein carrying the single-Bβ epitope (BEP-TolA-Avi) as well as a control protein lacking Bβ epitope (ΔEP-TolA-Avi) were also constructed and produced as above. Synthetic peptide CNIPVVSGKECEEIIR (sBEP) was produced by Vidia s.r.o. (Vestec, Czech Republic).

### 2.2. Ribosome Display Selection of Binders

Combinatorial DNA library was generated as described previously [33,34] with some modifications. The assembled library was in vitro transcribed/translated in a single step reaction using *E. coli* extract (EasyXpress *E. coli* kit, biotechrabbit, Hennigsdorf, Germany) and used for the pre-selection in 96well Maxisorp plates (NUNC, Roskilde, Denmark). To reduce non-specific variants, two pre-selection steps were performed (each 1 h at 4 °C): first one on fibrinogen and the second on the ΔEP-TolA-Avi. Fibrinogen (Abcam, Cambridge, UK) was coated to the wells of plate directly at concentration of 5 µg/mL for all three rounds of the selection. The biotinylated protein ΔEP-TolA-Avi was coated indirectly (10, 10 and 2.5 µg/mL for individual rounds) via streptavidin (1 µg/mL in carbonate buffer pH 9.6). The selection of binders was made in wells with 3BEP-TolA-Avi (10, 10 and 2.5 µg/mL for individual rounds) bound via biotin to coated streptavidin (1 µg/mL in carbonate buffer pH 9.6). After 1 h incubation at 4 °C, selection well was in the washed 5 times (10 times in second and third round) with wash buffer (50 mM Tris, 150 mM NaCl, 50 mM Mg-acetate, pH 7.5) supplemented with 0.05% Tween 20 (0.05% and 0.25% for second and third round, respectively). Collection of cDNA, obtained by reverse transcription after the third round of selection campaign, was cloned as NcoI and BamHI fragments in a pET-28b-TolA-Avi vector containing DNA sequences for spacer TolA and C-terminal Avitag [34]. The final TolA-Avi fusion proteins were expressed in *E. coli* BL21 (λDE3) GOLD strain. Whole cell lysates of individual clones were used for ELISA screening of binding to fibrin.

### 2.3. Binding of Protein Variants to Fibrin

Fibrin was formed directly in wells of Maxisorp 96-well plate from coated fibrinogen (10 µg/mL) by incubation with 0.001 U of thrombin (Abcam, Cambridge, UK) in the reaction buffer (50 mM Tris pH 7.4, 150 mM NaCl, 10 mM CaCl_2_ and 7 mM cysteine where stated) overnight at room temperature. After washing three times with PBST buffer (phosphate buffered saline with 0.05% Tween-20) and blocking with 1% bovine serum albumin (BSA) in PBST (PBSTB), cell lysates or serially diluted protein binders in PBSTB buffer were applied. Detection was made by mouse anti-Avitag antibody (antibodies-online, Aachen, Germany) followed by anti-mouse horseradish peroxidase (HRP) conjugated secondary antibody or by streptavidin-HRP conjugate in case of detection of biotinylated protein. TMB-Complete 2 solution (TestLine Clinical Diagnostics s.r.o., Brno, Czech Republic) was used as a substrate for HRP. Reactions were stopped with 2 M sulfuric acid and absorbance was read at 450 nm. As a control, simultaneous binding to fibrinogen was also monitored.

### 2.4. Production and Purification of D7/E7-TolA-Avi Protein

*In vivo* biotinylated D7 and E7 proteins in fusion with TolA-Avi was produced in *E. coli* BL21 (DE3) cells with inserted vector bearing *BirA* gene coding biotin ligase. Upon induction of the culture, the biotin was added and the protein was in vivo biotinylated on the C-terminal Avitag. N-terminal His-tag was used for a native purification on Ni-NTA agarose matrice.

### 2.5. Preparation of Layers of Fibrin Degradation Products and Fibrinogen

MaxiSorp 96-wells flat-bottom microtiter plate was used for the immobilization of anti-human fibrinogen goat antiserum (30%) (Kamiya Biomedical Co, Seattle, WA, USA). After 1 h incubation of 100 µL of anti-fibrinogen antiserum (1:500 dilution of stock solution) wells were washed out by 200 µL 50 mM Tris, 100 mM NaCl pH 7.4 with 0.1 % Tween-20 (TB-T) for 10 times. Tween-20 was used for minimalizing of non-specific binding (5% solution; 100 µL; 1 h incubation). Wells were then washed out by 200 µL TB-T for 10 times. The next step was binding of 100 µL fibrinogen (FGL, FGP) or fibrin degradation products (FBL, FBP) for 1 h that were prepared freshly before the experiment. Lyophilized fibrinogen (Sigma-Aldrich, St. Luis, MO, USA) was used as a source for preparation of FGL and FBL, while pooled plasma was used for production of FBP and FBP production. Pooled plasma was produced by mixing twelve plasma samples from healthy donors. To prepare FBL, 1 mL of FBG (2 mg/mL) was mixed with 10 µL FXIIIa (1% solution) and 190 µL thrombin (65 U/mL), followed by 2 h incubation. Then, the formed thrombus was digested by plasminogen (0.15 U/mL) and tissue plasminogen activator (tPA, 0.3 µg/mL) in 300 µL TB during further 2 h incubation. For FBP preparation, 1 mL of pooled plasma was mixed with 190 µL thrombin (65 U/mL) and 10 µL CaCl_2_ and incubated for 2 h. Then, the formed thrombus was digested by plasminogen (0.15 U/mL) and tissue plasminogen activator (tPA, 0.3 µg/mL) in 300 µL TB during further 2 h incubation. Preparations FBL and FBP were used in preparation of ELISA plates above. FGL and FGP as controls were incubated only with 500 µL of TB.

### 2.6. Binding of Protein Variants to Fibrin Layers Prepared from Lyophilized Fibrinogen or Human Plasma

Wells of 96-well plates coated with fibrin/fibrinogen layers were washed out with TB-T buffer and non-specific interactions were blocked out by 1 h sorption of serum bovine albumin (1%). After washing out of wells, 100 µL of protein binders (D7, E7; 1 µM) or mouse anti-fibrinogen antibody (1:1000; American Diagnostica GmbH, Pfungstadt, Germany) were added for 1 h incubation. For detection of binders or antibody, ExtrAvidin (100 µL, 1:20000; Sigma-Aldrich, St. Luis, MO, USA) or anti-mouse IgG (100 µL, 1:30000; Sigma-Aldrich, St. Luis, MO, USA) conjugated with alkaline phosphatase were used, respectively (1 h incubation). Para-nitrophenylphosphate (6 M) was used as a substrate for alkaline phosphatase. The change of optical properties was monitored by Sunrise ELISA Reader at 405 nm.

### 2.7. Construction of Truncated D7 Proteins with C-Terminal His-Tag

To produce D7 protein version lacking the TolA spacer and containing His-tag at the C-terminus, a trp leader sequence consensus (MKAIFVLNAQHDEAVDAMD) was introduced to the N-terminus. Then, a FLAG/His-tag in a tandem was attached to the C-terminus via 9- or 21-amino acid long GS-linker. The calculated Mw of all products is 11–12 kDa. The production of these proteins was performed in *E. coli* BL21 (DE3) cells at 37 °C.

### 2.8. Binding of D7 Protein Variants to Human Thrombus

Thrombus preparation. Human blood was donated by healthy volunteers (*n* = 10) who signed an informed consent. The informed consent was approved by the ethical committee of the International Clinical Research Center St. Anne’s University Hospital Brno on 2017-03-07 (IIT/2016/30). The blood donors did not take any medication at least two weeks before blood collection.

Plasma Thrombus Preparation. Citrated blood (100 µL per 10 mL of blood) was centrifuged at 2000 g to obtain plasma. Plasma (50 µL) was coagulated in 8 mm glass tubes with addition of 10.9 mM CaCl_2_ and NaCl and 50 mM thrombin at room temperature for 4 h. Each thrombus was cut into four pieces. Before a start of an experiment each thrombus was gently washed with 2 mL of TBS buffer.

Preparation of thrombi from whole blood. Thrombi were prepared from 10 µL of whole blood without addition of anticoagulants in glass tubes—Target Insert 300 µL Capacity (National Scientific Supply Company Inc., Claremont, CA)—at room temperature for 4 h. Before a start of an experiment each thrombus was gently washed with 2 mL of TBS buffer.

Confocal microscopy of D7-TolA-Avi and *His-tagged D7* targeted fibrin filaments. D7-TolA-Avi (final concentration of 20 µg/mL) was incubated with prepared thrombus in TBS buffer for 30 min. After that, each thrombus was washed twice by 200 µL of TBS buffer and placed into TBS buffer with 6.6 µg/mL APC streptavidin (BioLegend, San Diego, CA, USA) for 60 min. Each thrombus was washed three times with 200 µL of TBS buffer prior to observation using confocal microscope Leica TCS SP8. Excitation and emission wavelengths were set to 633 and 645–700 nm, respectively. For visualization of His-tagged D7 variants (containing mono His-tag and Flag-tag or double His-tag) anti-Flag-tag (Monoclonal ANTI-FLAG^®^ M-Cy3^TM^, Clone M2, Sigma-Aldrich, Prague, Czech Republic) and anti His-tag (Alexa Fluor^®^ 488 anti-His Tag Antibody, BioLegend, San Diego, CA, USA) antibodies were used, respectively. The concentration was 10 µg/mL and the incubation lasted one hour.

### 2.9. Preparation of D7 Liposomes Modified by Different Variants of Anti-Fibrin Protein Binder D7

Briefly, metallochelation liposomes were prepared by a method based on a hydration of a lipid film followed by extrusion through 0.4 μm and 0.1 μm polycarbonate filters as described previously [35,36] Liposomes were composed of EPC/DOGS-NTA-Ni/ Liss Rhod PE, 92.5/7.0/0.5 molar% *w*/*w*/*w*. All lipids were purchased from Avanti Polar Lipids ((Alabaster, AL, USA). A manually operated device Mini-Extruder (Avanti Polar Lipids) was used for the extrusion. In the following step, the solution of D7 protein variants of binder in 50 mM Tris, pH 7.4 was mixed with the prepared solution of liposomes. The protein/lipid ratio was 1/20 *w*/*w*. The mixture was incubated for 30 min at room temperature.

### 2.10. Characterization of D7 Liposomes

Size measurement using the multiangle dynamic light scattering technique (MADLS). The hydrodynamic diameters of the binders, liposomes and proteoliposomes were determined by the MADLS technique using Zetasizer ULTRA instrument (Malvern Panalytical, Malvern, UK) at 25 °C.

Isotermal titration of liposomes by D7 protein binders. Calorimetric measurements were carried out to determine dissociation constants and thermal effects occurring during the process of formation of metallochelation bond between His-tag of protein binder and Nickel ion of DOGS-NTA-Ni lipid incorporated into liposomes. Gradual addition of the protein binder solution from a syringe with a volume of 40 μL to the titrated liposomal solution (0.5 mg/mL) with a volume of 350 µL located in a cell was performed using MicroCal PEAQ calorimeter (Malvern Panalytical, Malvern, UK). Experiments were performed in 50 mM Tris buffer, pH 7.4 at a temperature of 25 °C. The concentration of different variants of D7 protein solution was adjusted individually. The mixing rate was 750 rpm during the titration.

Transmission electron microscopy. The suspension of liposomes was covered with a Cu grid (300 Old Mesh, Agar Scientific, Austria) coated with Formvar film (Sigma Aldrich, Czech Republic) and carbon. The grid was removed from the suspension after 1 min, and the residual water was dried with a strip of lint-free filtration paper. The sample we stained with 2% Ammonium molybdate and observed under transmission electron microscope Philips 208S Morgagni (FEI, Czech Republic).

Immunogold technique was employed to prove specific binding of D7 to the surface of liposomes. Anti-FLAG antibody and 20 nm protein A colloidal gold conjugate (Electron Microscopy Science, Hatfield, PA, USA) were added to D7 liposomes and observed using transmission electron microscopy for specific staining of protein binder on the surface of liposome. The solution of Protein-A covered gold nananoparticles were diluted in ratio 1:50 and mixed with liposomal samples in the volume ratio 1:1. The mixture was incubated overnight at 4 °C and observed.

### 2.11. Confocal Microscopy of Thrombi Targeted with D7 Liposomes

Rhodamine-labeled D7 targeted liposomes (final concentration of 15 µg/mL) were incubated with prepared thrombus in TBS buffer for 30 min. After that, each thrombus was washed five times with 200 µL of TBS buffer prior to observation using confocal microscope Leica TCS SP8. Excitation and emission wavelengths were set to 561 and 580–650 nm, respectively.

### 2.12. Scanning Electron Microscopy of Whole Blood Thrombi

Samples for the scanning electron microscopy of whole blood thrombi or thrombi with fibrin-targeted liposomes were fixed in Millonig phosphate buffered gluteraldehyde (3%), post-fixed in osmium Millonig buffered (OsO4 2%) solution, dehydrated in 50, 70, 90, and 100% Ethanol and dried in HMDS (hexamethyldisilazane, Sigma-Aldrich, Prague, Czech Republic). Then the samples-were put on the carbon tabs attached on the aluminum holder and platinum/palladium coated (Cressington sputter coater 208 HR, Watford, UK). The surface of the thrombus was observed under scanning electron microscope Hitachi SU 8010 (Hitachi High Technologies, Europe GmbH, Krefeld, Germany) at magnification of 2000× (at 14 kV, SE detector, working distance 10.8 mm).

### 2.13. In Vitro Binding of D7F1 Liposomes under Flow Conditions Using MCA Model

Simplified virtual middle cerebral artery (MCA) models with a branch (bifurcation) were designed with Fusion 360 (Autodesk) with anatomy resembling average middle cerebral artery diameter 3.1 mm, (after bifurcation 2.9 mm, the branch 1.4 mm). The lumen was three-dimensionally (3D) printed and cast into silicone Sylgard^®^ 184 Elastomer kit (Dow Corning, Bay City, MI, USA). The silicone model was connected to a peristaltic pump Lambda Multiflow (Lambda Laboratory Instruments, Brno, Czech Republic) with 3.1 mm (inner diameter) tubes and filled with TBS buffer (pH 7.4). Externally prepared in vitro clot from whole blood (100 µL a diameter of each glass tube was 6 mm for 100 µL of blood.) was inserted into tubing through a funnel to simulate the thromboembolic event in MCA. After MCA occlusion the buffer was flowing through a branch at 4.5 mL per minute. D7F1 liposomes were injected by Hamilton syringe into the flow system. The TBS buffer with final concentration of 15 µg per ml D7F1 liposomes was circulating one hour before the clot inside the in vitro model was extracted from the in vitro model, washed with TBS and examined under confocal microscope (Leica) to observe penetration of fluorescently labeled liposomes into the thrombus.

## 3. Results

### 3.1. Production of Recombinant Protein Targets Carrying Bβ Epitope (BEP)

**Figure 1:**
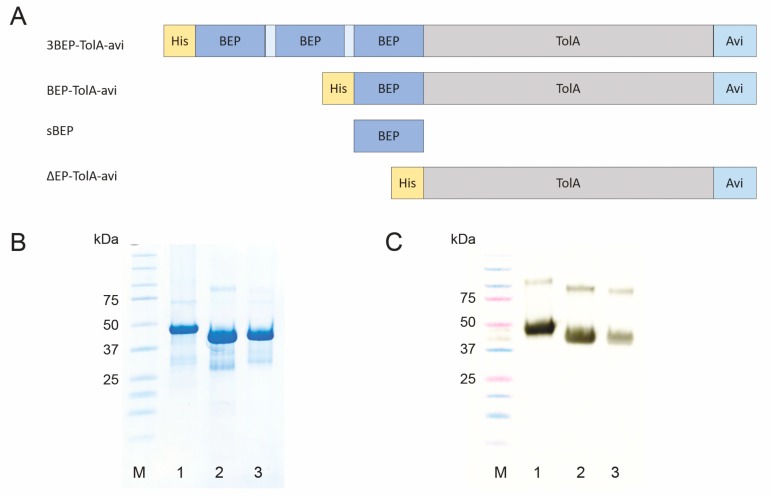
Schematic representation of BEP-carrying targets and their production and identification. In vivo biotinylated proteins carrying triple BEP epitope (3BEP-TolA-Avi), single BEP epitope (BEP-TolA-Avi), 16 amino acid synthetic BEP peptide (sBEP) and a control BEP-lacking protein (ΔBEP-TolA-Avi) shown in panel **A**. Visualization of produced proteins on sodium dodecyl sulphate-polyacrylamide gel electrophoresis (SDS-PAGE): molecular weight marker (lane M), 3BEP-TolA-Avi (lane 1), BEP-TolA-Avi (lane 2), ΔBEP-TolA-Avi (lane 3), respectively (panel **B**). Western blot of purified recombinant proteins (panel **C**) detected by streptavidin-HRP conjugate. Description of lanes is as presented in panel B.

In this study, a short protein corresponding to Bβ chain epitope of human fibrinogen recognized by 102-10 mAb (BEP) as a molecular target for the ribosome display was used. This epitope discovered by Hisada et al. [16] was predicted to be sterically shielded in fibrinogen but uncovered and exposed during fibrin thrombus formation. This epitope consisting of 16 amino acid residues (CNIPVVSGKECEEIIR) was, therefore, chosen as a major N-terminal binding motif for design and assembly of the recombinant protein, constructed in fusion with C-terminal Avi-tag. To facilitate protein purification, the sequence of His-tag was added to the N-terminus of the full-length construct (BEP-TolA-Avi, Figure 1). To significantly expose BEP moiety as a target for ABD screening, and to increase changes for the recognition during ribosome display, a variant carrying triple-BEP motives connected via GGGGS-linkers (3BEP-TolA-Avi) was also designed and assembled. As a control, protein lacking BEP epitope (ΔBEP-TolA-Avi) was also generated. All proteins were produced in *E. coli* BL21 cells and subsequently purified (Figure 1).

### 3.2. Ribosome Display and Screening of Protein Variants

**Figure 2:**
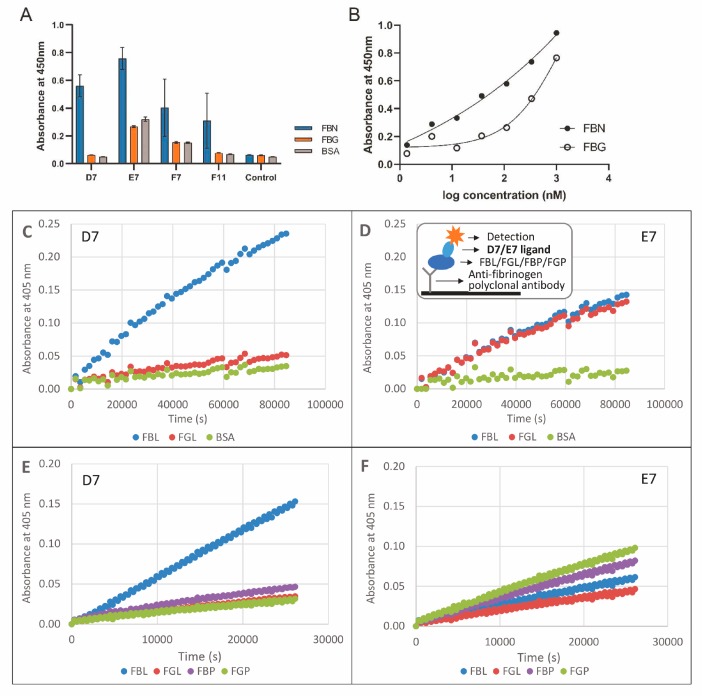
Binding of D7 and E7 protein variants to fibrin layer tested by ELISA. Binding of *selected* in vivo biotinylated protein variants to immobilized fibrin, fibrinogen and bovine serum albumin detected by streptavidin-HRP conjugate. Average values of triplicates with standard deviations are presented (**A**). Binding curves of in vivo biotinylated D7-TolA-Avi protein to fibrin (FBN) and fibrinogen (FBG) in ELISA (**B**). Binding of D7-TolA-Avi (**C**,**E**) and E7-TolA-Avi (**D**,**F**) protein variants to fibrinogen and fibrin layers tested by ELISA. Binding of protein variants to layers prepared from lyophilized fibrinogen (**C**,**D**) and those prepared from human plasma (**E**,**F**). Insert in the panel D shows a schematic representation of fibrin (FBN)/fibrinogen (FBG) layers and detection of the bound proteins. Legend: FBN—fibrin, FBG—fibrinogen, BSA—bovine serum albumin, FBL—fibrin layer prepared from lyophilized fibrinogen activated by thrombin, FGL—layer of fibrinogen produced from lyophilized product, FBP—fibrin layer prepared from human plasma activated by thrombin, FGP—layer of fibrinogen from human plasma.

For ribosome display, assembled and purified 3BEP-TolA-Avi protein was chosen as a target. To minimize selection of binders raised to a TolA-Avi protein backbone, a preselection step using the generated ΔBEP-TolA-Avi protein was used. After a three-round ribosome display campaign with an increasing stringency of the selection conditions, transcribed cDNA sequences were inserted into the pET-28b plasmid, thus generating a library of the binding candidates. Cell lysates of individual bacterial colonies were screened for binding to coated fibrin and fibrinogen by ELISA. Protein variants with a substantial binding to fibrin and that reduced to fibrinogen were selected, their proteins purified and further examined in a detail. From the collection of almost 400 tested clones, four variants named D7, E7, F7 and F11 confirmed the preferential binding to the insoluble fibrin and were, therefore, selected as the most promising ones (Figure 2A, Table 1). Sequencing of the particular cDNA cloned into the plasmid vector revealed that E7 and F7 variants are identical. Further testing demonstrated a high binding of E7 and F11 proteins to the soluble fibrinogen. D7 protein was, therefore, chosen as the only suitable binder with a substantial binding preference to insoluble fibrin and its binding curve was measured by ELISA (Figure 2B).

### 3.3. Binding of D7 and E7 Protein Variants to Fibrin Layer

After ribosome display, a large-scale screening of the binding variants was performed using thrombin-treated fibrinogen coated on wells of microtitre plates. To verify that the identified D7 and E7 binding candidates bind not only to a coated insoluble fibrin but also to fibrin degradation products, tests on layers of fibrin and fibrinogen captured to the plate via polyclonal antibody (Figure 2C,D) were performed. These experiments confirmed that D7 clone preferentially binds to fibrin while binding to fibrinogen is minimized and stays on the background level. Contrary this, E7 protein variant does not distinguish between fibrinogen and fibrin and binds to both proteins in a similar way. Additionally, we compared binding of D7 and E7 variants to fibrin layer prepared from a commercial lyophilized fibrinogen or from isolated human plasma (Figure 2E,F). We found that the binding to fibrin prepared from a lyophilized product is more pronounced than binding to fibrin produced from human plasma. In this experiment, E7 protein demonstrated its high binding to lyophilized fibrinogen as well as to human plasma.

### 3.4. Binding of D7 Protein to Human Thrombus in Vitro

**Figure 3:**
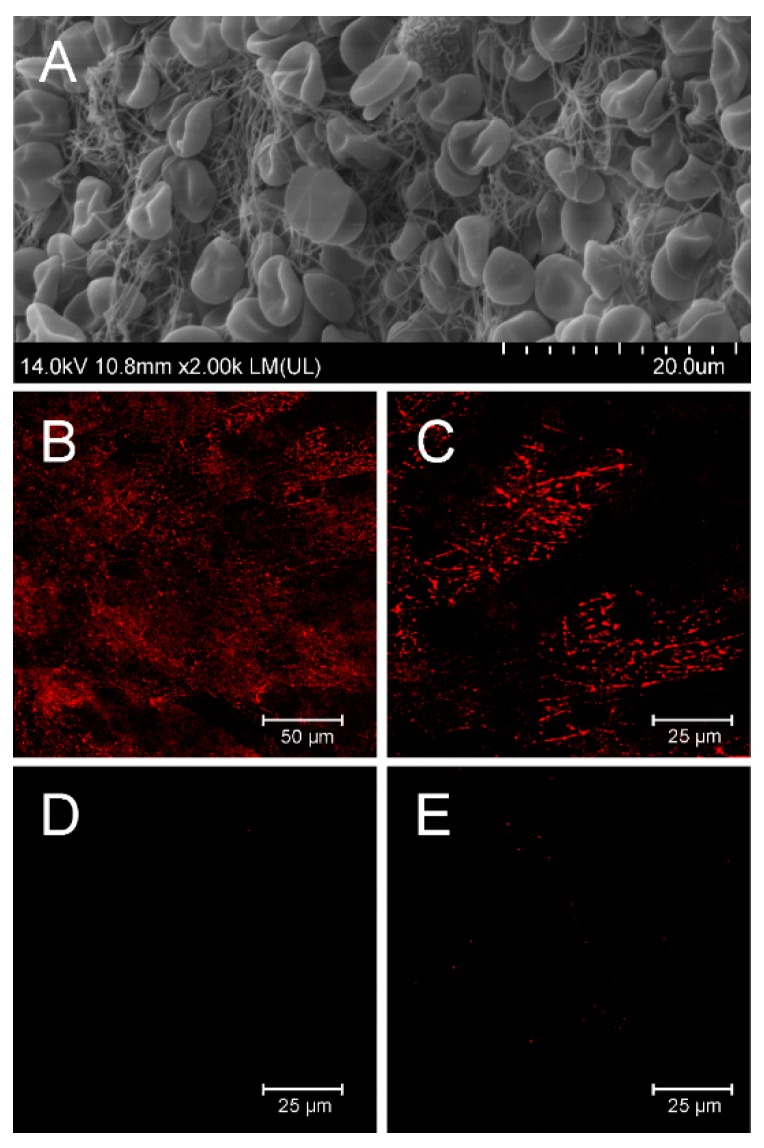
In vitro binding of fibrin filaments in the human whole blood thrombus by D7-TolA-Avi. Quality control of the structure of prepared whole blood thrombi was confirmed by SEM. Representative picture of thrombus prepared for binding experiments with a clear structure of fibrin filaments (**A**). D7-TolA-Avi was tested for the ability to target fibrin filaments of the human blood clots. APC-streptavidin was used for visualization. Samples were observed using Leica TCS SP 8 confocal microscope (excitation 633 nm, emission 645–700 nm). The representative picture illustrates the specific interaction of D7-TolA-Avi with fibrin in human blood thrombus (**B**). Detailed picture of fibrin in the blood thrombus. Non-homogeneous distribution of the signal is given by the observation of a thin confocal plane (**C**). Negative control—picture of thrombus incubated with non-specific ABDwt-TolA-Avi protein and APC-streptavidin (**D**), and a negative control—picture of thrombus incubated with APC-streptavidin (**E**).

To verify whether D7-TolA-Avi recognizes fibrin filaments in human thrombus, binding of the D7 protein to thrombus prepared from human whole blood was visualized by confocal microscopy (Figure 3). The quality of the prepared thrombus is documented in the panel A and detection of clearly visible fibrin fibers by D7-TolA-Avi protein is presented in the panels B and C. Negative staining by a control ABDWT-TolA-Avi protein (D) or in the presence of secondary reagent only (E) are also presented.

### 3.5. Production of Short Variants of D7 Binding Protein with C-Terminal His-Tag

**Figure 4:**
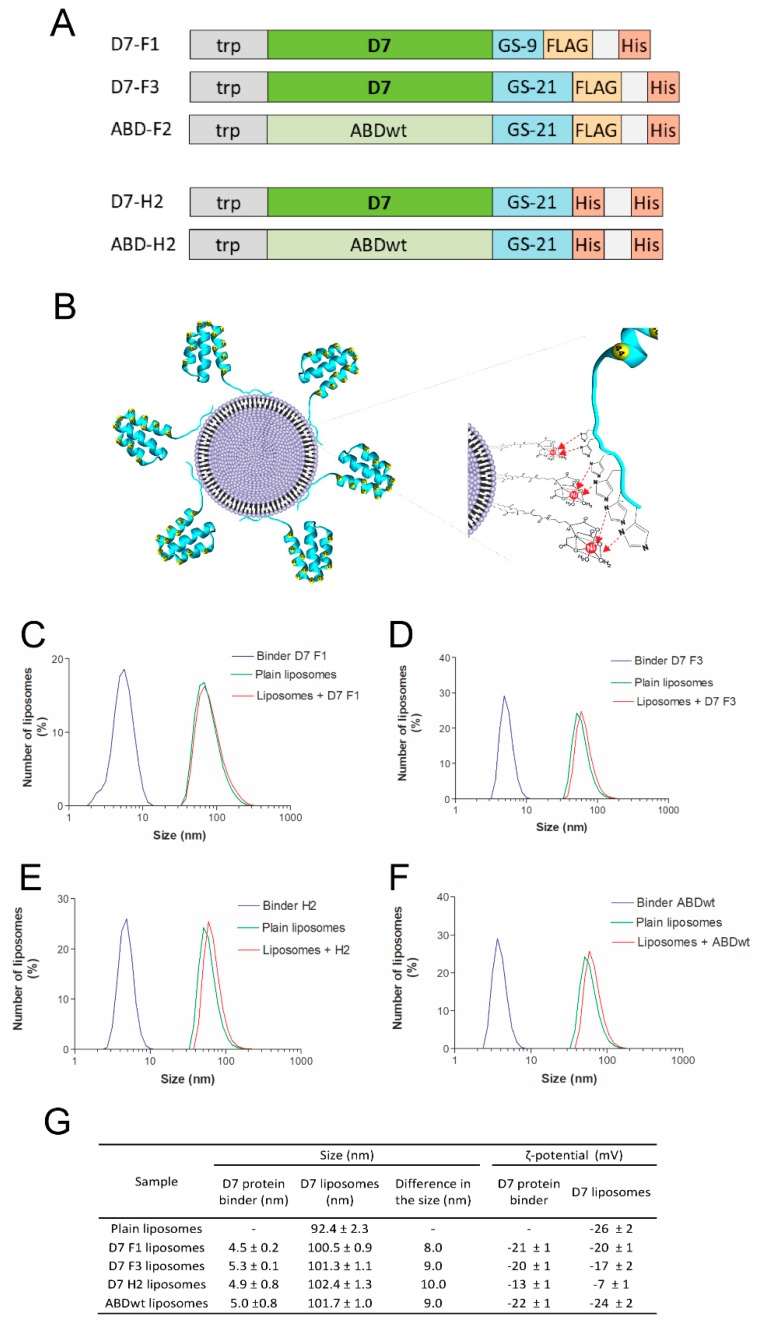
Characterization of liposomes modified by ABD-protein binders using MADLS. Schematic representation of C-terminally polyhistidylated variants of D7 binding proteins and ABD-WT control (**A**). Schematic representation of metallochelation bond of polyhistidylated variants of D7 proteins onto the surface of liposomes (**B**). The size distribution of mono- and double-His-tagged binders, plain liposomes and proteoliposomes was measured using MADLS technique. Increase in the size of plain liposomes followed its surface modification with both mono- and double-His-tagged binders was observed. Size distribution of D7F1 (**C**), D7F3 (**D**), D7H2 (**E**) and ABDwt (**F**) modified liposomes are shown. Inserted table summarizes the change in the size and ζ-potential followed liposome modification (**G**).

For the purpose of an immobilization of the D7 protein to nanoliposomal particles via interaction of a polyhistidylated tag with Ni-NTA-modified surface, truncated proteins were designed and produced from plasmids in which D7 protein cDNA was fused to GS-linker with FLAG-tag and His-tag at the C-terminus (Figure 4A). The D7-F1 protein contains a shorter GS-linker in comparison to the D7-F3 version (nine versus 21 amino acids, respectively). To increase binding affinity to Ni-NTA-modified liposomal surface, we constructed also double-His-tag versions of the truncated D7 and ABD-WT proteins using the same GS-linker as that used for FLAG-His variants preparation (Figure 4A).

### 3.6. Preparation and Characterization of Liposomes

Plain metallochelating nanoliposomes (EPC/DOGS-NTA-Ni 93/7 *w*/*w*) of the size about 92 nm and negative ζ-potential were prepared by lipid hydration method and extrusion (Figure 4). Selected specific binders (Figure 4A) as well as their control counterparts were bound onto liposomal surface via metallochelating interaction (Figure 4B). This binding was reflected by an increase of hydrodynamic radius of formed proteoliposomes. The increase in the size of modified liposomes was in a good accordance with hydrodynamic radius of various binders used (Figure 4C–F). Binders itself possess negative charge and modification of the liposomal surface with binders did not change significantly the negative ζ-potential of liposomes, but only in the case of binder D7H2 a shift to positive value of ζ-potential was observed (Figure 4G).

**Figure 5:**
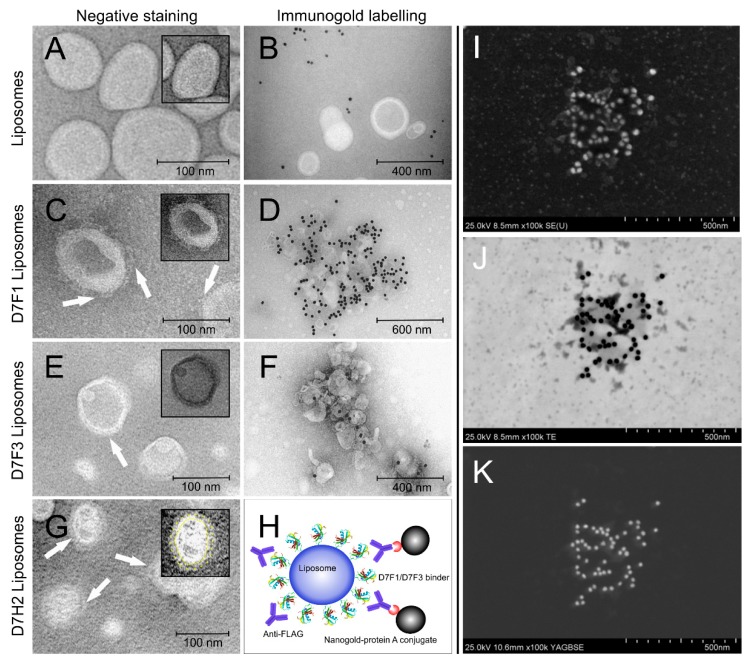
TEM of liposomes modified by mono His-tagged D7F1, D7F3 and double His-tagged DFH2 variants of ABD-protein binders using negative staining and immunogold labeling technique. Contrast of liposomes was enhanced using negative staining with exception of H-J. TEM micrographs are displayed of plain metallochelation liposomes (**A**), D7F1 (**C**), D7F3 (**E**) and D7H2 proteoliposomes (**G**). Binders are clearly visible on the surface of all binder-modified liposome samples. Immunogold labeling using anti-FLAG M2 antibody was used for specific detection of binder attached to the surface of metallochelation liposomes: control plain liposomes (**B**), D7F1 liposomes (**D**), D7F3 (**F**). Schema representing specific detection of binder molecules using the system of Anti-FLAG antibody and 20 nm protein A colloidal gold conjugate. (**H**–**K**) Detail of liposomes with binder D7H2 visualized by specific immunogold staining and detected also by using TEM equipped with SE, TE, YAGBSE detectors, respectively. Insets in (**A**,**C**,**E**,**G**): highlighted structure of selected proteoliposomes by image over contrasting, color inverting and/or labeling clearly show a protein corona formed by protein binders on the surface of liposomes.

Modification of liposomal surface by binders was also confirmed by TEM and immunogold TEM. Comparison of TEM pictures of plain and binder modified liposomes demonstrated direct visualization of binder molecules on the liposomal surface (Figure 5A,C,E,G). The presence of binder molecules on the surface of nanoliposomes was confirmed by immunogold staining technique. Gold nanoparticles modified with anti-FLAG antibodies recognized the protein binders on the liposomal surface and liposomes were labeled with contrast gold nanoparticles, while the plain liposomes were not labeled (Figure 5B,D,F). Figure 5H represents the schematic picture of specific binder staining directly on the surface of liposomes. The visualization of gold nanoparticles on the surface of D7 liposomes was done by different detectors (Figure 5H–J)—SE, TE and YAGBSE detectors, respectively.

### 3.7. Thermodynamic Characterization of Interaction between ABD-Protein Binders and Metallochelation Liposomes

Isothermal titration experiments enable direct measurement of Δ°G and calculation of dissociation constant Kd for binder-liposome complexes prepared at 25 °C (298.15 K). Binders with double His-tag exerted lower dissociation constant in comparison to binders with one His-tag in their molecule. This means that insertion of double His-tag increased the binding of protein binders onto liposomes. Results are summarized in Table 2.

### 3.8. Interaction of Fibrin-Specific Binders with Thrombi

**Figure 6:**
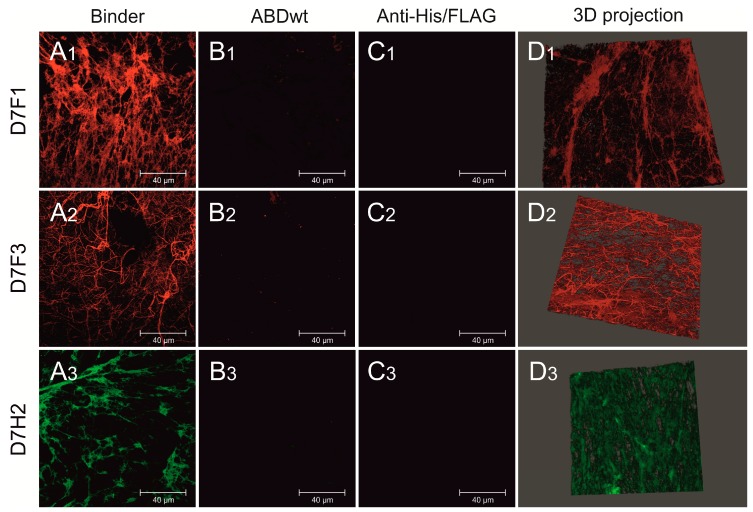
Confocal microscopy of fluorescently labeled D7F1, D7F3 and DFH2 variants of ABD-protein binders attached to fibrin fibrils of human thrombus. Various ABD-protein binders were incubated with human thrombus, washed and visualized using fluorescently labeled antibodies. D7F1 and D7F3 variants of ABD-protein binders were visualized using Anti-FLAG^®^ M2 Cy3 Antibody Conjugate (colored in red—**A1**,**A2**). D7H2 binder was visualized using Anti-6X His tag^®^ Alexa Fluor^®^ 488 antibody (colored in green—**A3**). Non-specific ABDwt was used as a negative control (**B1**–**B3**). Anti-FLAG^®^ M2 Cy3 Antibody Conjugate and Anti-6X His tag^®^ Alexa Fluor^®^ 488 antibody were incubated with thrombus without the presence of fibrin-specific binders as a negative control (**C1**–**C3**). 3D reconstruction of stained fibrin meshes (**D1**–**D3**). Displayed scale bar 40 µm.

To confirm the ability of selected binders to specifically bind to human thrombus (binder variants containing one His-tag (D7F1, D7F3) and two inserts of His-tag (D7H2), fibrin fibrils in thrombi was visualized by confocal microscopy after the incubation with fluorescent conjugates (Figure 6(A1–A3)). All tested binders exerted strong binding onto fibrin in thrombi in comparison to their wild-type counterparts, which did not bind the fibrin at all (Figure 6(B1–B3)). Fluorescent conjugates (anti-FLAG and anti-His-tag) without the incubation with binders were used as controls (Figure 6(C1–C3)). Structure of fibrin thrombus with bound binders stained with fluorescence anti-FLAG and anti-His-tag antibodies is visualized in 3D projection (Figure 6(D1–D3)). In addition, the ability of D7F1 to bind to the fibrin fibrils of mouse thrombus was tested according the same protocol. Although the observed signal was significantly lower as compared to human thrombi, the ability of binding onto fibrin fibers was confirmed (supplementary file, Figure S1).

### 3.9. Interaction of Targeted Liposomes with Thrombi

**Figure 7:**
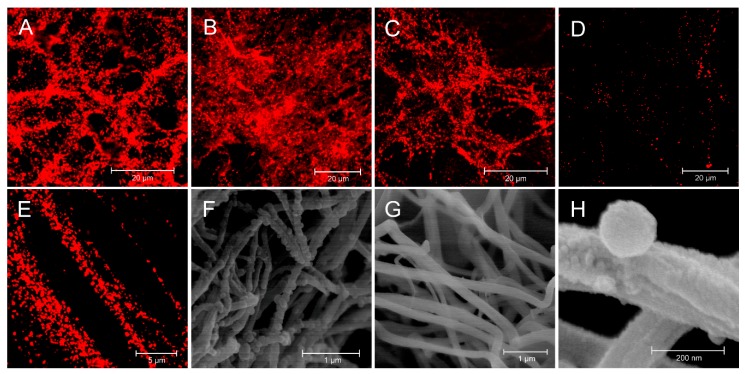
Binder-modified proteoliposomes targeted to fibrin fibers of human thrombus. Confocal microscopy and SEM were used for visualization of binder-modified proteoliposomes bound to the fibrin fibrils of human thrombus. Confocal microscopy of D7F1 (**A**), D7F3 (**B**) and D7H2 (**C**) modified liposomes labeled with fluorescent probe rhodamine-lyssamine PE. Fluorescent plain liposomes were used as control (**D**). Detailed picture demonstrate the binding of D7H2 liposomes onto the surface of fibrin fibrils using confocal microscopy (**E**). SEM—picture of fixed thrombus with attached D7H2 proteoliposomes (**F**) with its negative control (**G**). Detail of direct interaction between D7H2 liposome and surface of fibrin fiber (**H**).

Fibrin-selective binders D7F1, D7F3 and D7H2 were bound onto the surface of metallochelating nanoliposomes labeled with fluorescence probe Rhodamine-Lyssamine PE via formation of His-tag—Nickel complexes. Binding of liposomes targeted by various types of binders onto particular thrombi was visualized by confocal microscope. Liposomes targeted by selective binders D7F1, D7F3 and D7H2, respectively, were able to bind fibrin fibers and fibrous structure of the thrombi was clearly imagined by confocal microscopy (Figure 7). Massive binding of targeted liposomes was observed (Figure 7A–C). It is interesting that plain liposomes also adhered onto fibrin fibers, but the number of bound liposomes was lower in comparison to the targeted ones (Figure 7D). Detail of interaction of D7H2 liposomes with fibrin fibers is visualized in Figure 7E. SEM picture of fixed thrombus with bound D7H2 liposomes and its negative control are in Figure 7F,G, respectively. Detailed visualization of direct interaction between liposome and surface of fibrin fiber is displayed in the Figure 7E.

### 3.10. In Vitro Model of Thrombus Obstruction in Artery for the Evaluation of in Vitro Binding of Liposomes onto the Fibrin in Thrombi

**Figure 8:**
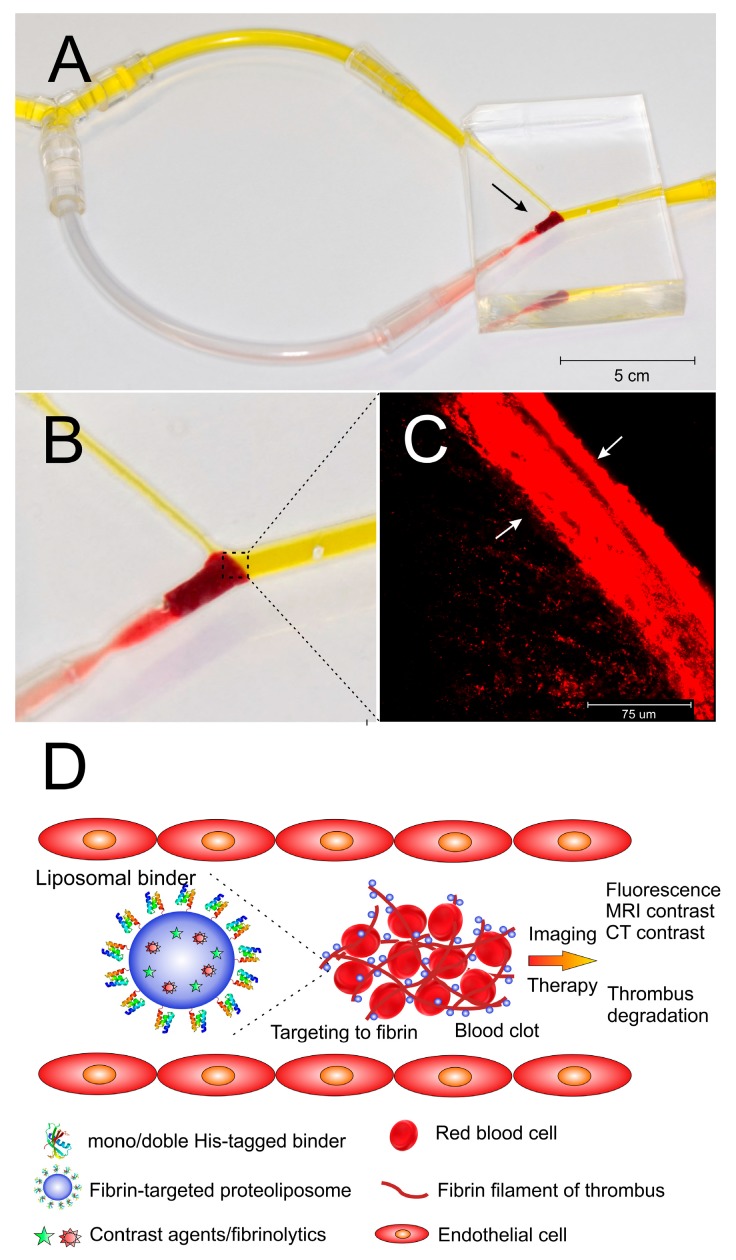
In vitro binding of targeted nanoliposomes to the thrombus under flow conditions. Picture of silicone replica of middle cerebral artery and its connection to the fluid circulation drown by flow pump. The site of the occlusion caused by introduced thrombus is marked by the arrow (**A**). Detailed view of the site of occlusion (the circulation of fluid is highlighted by yellow color) (**B**). Penetration of fibrin-targeted liposomes to the thrombus withdrawn from the silicone model detected by fluorescence confocal microscopy. Depth of penetration is marked by arrows (**C**). Schematic picture of targeting of fibrin fibers of the thrombus under flow conditions (**D**).

Thrombus was placed in a silicone replica of the human middle cerebral artery to mimic the situation of complete occlusion of the artery (Figure 8A,B). In vitro binding of fibrin in the thrombus by fluorescently labeled liposomes was than examined under flow conditions to mimic possible real situation occurring *in vivo*. After 20 min medium was replaced with those free of liposomes and perfusion continued for next 10 min to remove non-bound liposomes. The thrombus was removed after the completing the experiment and confocal microscopy was used to prove the perfusion of targeted liposomes into the thrombus. Liposomes penetrated into thrombus and the depth of penetration was clearly detectable by confocal microscopy. After 20 min of the incubation of the thrombus in the model under flow conditions, the depth of massive penetration of D7H2 liposomes in the range of 50–100 µm was observed (Figure 8C).

## 4. Discussion

Fibrin formation is triggered by thrombin cleavage of fibrinopeptides on fibrinogen molecules, which allows them to spontaneously self-assemble into large fibers that provide the support structure of the thrombus and promote healing. Many aspects of their structure and functions still remain unknown [37,38]. The distinction between fibrinogen and fibrin is needed for determination of the size and place of thrombus formation. However, almost identical structure of fibrinogen and fibrin is hindering the process. Formation of insoluble fibrin is accompanied only by subtle changes in secondary structure and formation of very limited number of distinct new covalent bonds with new epitopes emerging. Presence of available free cysteine during polymerization process affect FXIIIa activity and possibly leads to remodeling of inter/intra molecular disulphide bonds resulting in new recognizable structures [39,40]. Interestingly, mouse and rat anti-insoluble fibrin monoclonal antibodies used in this study also significantly bind to fibrin formed in the presence of cysteine (supplementary file, Figure S2), while a diminished binding was observed in the absence of cysteine during fibrin formation.

In this work, we generated insoluble fibrin-targeted protein binders useful for development of thrombus imaging agents. For this non-immunoglobulin approach, combinatorial library derived from 5.5 kDa albumin-binding domain of streptococcal protein G was chosen as this library of theoretical complexity up to 10^14^ protein variants has already provided binders of several important protein targets [31,34,41], some of them with nanomolar or even sub-nanomolar binding affinity [30,32,33]. To stabilize small protein binders, 305 amino acid helical TolA protein is being used to form 38 kDa fusion binding variants. In combination with detection and purification tags, ABD-derived proteins can be simply modified by in silico and gene-fusion approaches. Therefore, ABD binders represent a suitable model for development of fibrin-targeted binders useful for development of thrombus imaging tools.

To target the insoluble form of fibrin, we used 16 amino acid peptide of the fibrinogen Bβ-chain (BEP epitope) [16] as an epitope for protein binder’s selection. This epitope, originally recognized by 102-10 monoclonal antibody reacting only with fibrin thrombus, was suggested as a unique region sterically shielded in fibrinogen or soluble fibrin, but conformationally changed and exposed in a polymerized form of insoluble fibrin fibers. Yet this feature has not been structurally defined nor independently confirmed, BEP-specific Fab fragment probe constructed from 102-10 mAb [18] was successfully used for tumor imaging in mouse model of pancreatic ductal adenocarcinoma (PDAC). Immunohistochemistry and ex vivo imaging confirmed selective distribution of the 102-10 Fab in fibrin thrombus in PDAC tumors with no observed effect on fibrinolysis or blood coagulation.

BEP is a conformation epitope and we, therefore, designed and constructed single and triple versions of BEP-derived fusion proteins as targets for ribosome display selection of anti-fibrin binders. To test function of these assembled proteins, we used mouse monoclonal antibody clone L (1101) and rat monoclonal antibody clone 443, both selected as BEP-specific and immunohistochemically verified in tumor cells. Both these antibodies confirmed a specific recognition of the 3BEP-TolA-Avi fusion in ELISA (Figure S3). In a striking contrast, control ΔBEP-TolA-Avi protein lacking ABD sequences, and also synthetic 16 amino acid peptide (sBEP), were not recognized by these antibodies. This result indicates the specificity of both antibodies for the BEP recognition, and as the linear peptide is not recognized by the used antibodies, this further supports a conformation requirement carried by 3BEP-TolA-Avi fusion protein. Thus, it can be used as a target for ribosome display in combination with a pre-selection step by ΔBEP-TolA-Avi. Interestingly, rat 443 as well as mouse 1101 mAb recognize 3BEP-TolA-Avi target regardless type of immobilization—by a direct coating or via C-terminal Avitag-mediated biotinylation (Figure S4). In addition, sensitivity/specificity of the BEP recognition is demonstrated by an increased signal of the triple-BEP protein in comparison to the single-BEP one.

Presented work relied on BEP-targeting approach for the selection of anti-insoluble fibrin binders. This strategy provided several binding candidates distinguishing between formed fibrin and the soluble fibrinogen. Based on tests of specificity, only D7 variant demonstrated a substantial preference for binding to fibrin in comparison to fibrinogen. Fibrin produced for in vitro large-scale screening of ABD variants, however, differs from that formed during a coagulation process *in vivo*. Fibrin polymer network produced in microtiter plates by thrombin has a limited cross-linking potential due to the immobilization to plastic substrate. Additionally, absence of factor XIII and plasmin may not produce D-dimers as in the case of blood thrombus formation. Despite these limitations, D7 protein substantially binds to fibrin layer prepared from lyophilized fibrinogen (Figure 2) similarly to binding of anti-insoluble fibrin monoclonal antibody (Figure S5).

A very interesting finding was that binding of D7 fibrin variants to fibrin prepared from a lyophilized product was different than binding to fibrin produced from human plasma. The influence of residual water on the solid-state properties of freeze-dried fibrinogen and dissolution of lyophilized fibrinogen studied by combined small- and wide-angle X-ray scattering (SWAXS) showed that there were differences triggered due to the different levels of residual moisture in various samples. The dissolution rates were found to decrease with increasing specific surface, most notably in the amorphous form, in contrast to expectations from classical thermodynamics. Protein conformational changes and hydrophobic surface formation upon depletion of water could be possible causes [42,43].

The most critical part of the development of anti-insoluble fibrin protein binders is to suppress fibrinogen recognition. The most specific variant identified after ribosome display selection, D7 protein, contains three proline residues randomized in positions 24P, 32P and 40P of the ABD scaffold structure. While amino acid residue 32 is located in a flexible loop between helix 2 and 3, position 24 and 40 can cause a local structural changes in the helices 2 and 3, respectively. Proline residues are known to change angles in helical structures, so modifications of the helical structure or reorientation of the helices in the D7 protein cannot be excluded. This can, however, support the fibrin Bβ epitope recognition and increase the observed anti-fibrin specificity. Beside recognition of human fibrin, confocal microscopy proved also selective binding of D7F1 binder towards fibrin in thrombi prepared from mouse blood. Binding of wild-type binder ABDwt as a negative control did exert negligible binding (see Figure S1).

Residual binding of D7 protein variant to fibrinogen, however, still remains substantial and needs to be suppressed. This can be done by generation of an optimized form of D7 protein by in silico docking using known structure of human fibrin D-dimer and structure of fibrinogen with the prediction of D7 protein mutations suppressing the fibrinogen recognition. Alternatively, molecular improvement of D7 variant can be performed by an affinity maturation approach using error-prone PCR-generated library and identification of variants with diminished binding to fibrinogen.

Non-covalent orthogonal binding of proteins with His-tag onto liposomes via metallochelating bond represent simple methods used for preparation of various proteoliposomes [35,44,45]. In our study, we tested protein binder constructs with one or two His-tags to compare complex stability constants (Table 2). As expected, double His-tag construct forms more stable complex with liposomes than constructs with one His-tag (Table 2). Changes of Gibbs energy differ in binders with one or two His-tag and this is reflected by the values of Δ°G and dissociation constants K_d_ of complexes. Relatively high stability of double His-tag (K_d_ in nM range) makes them suitable at least for various in vitro experiments designed to prove the concept and the value of K_d_ is comparable to that published for metallochelating complexes [46]. Doubling of His-tags seems to increase the stability of complexes in physiologic fluids and the measured nanomolar K_d_ value was sufficient for testing of thrombus detection under flow conditions. ABD-based D7-TolA-Avi binder demonstrated also sufficient thermal stability as shown in Figure S6 in supporting information. This characteristics is of interest for a possible future industrial-scale production and application.

Under flow condition in silicone replica of the human middle cerebral artery, the liposomes with D7H2 binder were able to penetrate through the mesh of fibrin fibers by diffusion and quickly bind to form the highly fluorescent zone at the forehead of thrombus. It is worth to note that behind the thrombus forehead, which is in the direct contact with the medium flow, the concentration of liposomes was significantly lower (Figure 8C). This, again, pointed to strong binding of binder targeted liposomes onto fibrin fibers. This is an important factor for future in vivo applications with respect to imaging of thrombi by MRI or CT, and for the thrombolysis mediated by liposome-delivered thrombolytic drugs (Figure 8D).

The mechanism of liposome penetration into thrombus revealed by in vitro model could have some consequences for in vivo solubilization of thrombus by application of targeted thrombolytic drugs like alteplase. The model predicts that the liposomes will move through the thrombus into its deeper part as a zone. It means that penetration of liposomes into thrombus will depend on concentration of liposomes in blood because they are trapped efficiently by fibers close to the forehead of thrombus washed with blood flow. Saturation of the surface of fibrin fibers in forehead of thrombus by liposomes enables the penetration of incoming liposomes deeper into thrombus by diffusion. Our experiments confirmed that nanoliposomes with the size around 100 nm are small enough to diffuse freely through the fibrin mesh of thrombi.

## 5. Conclusions

In summary, we present here the anti-insoluble fibrin binder D7 derived from the ABD scaffold. Small binding proteins are suitable for large-scale production and represent an innovative approach for the development of specific ligands that are important for targeting of drug nanocarriers such as nanoliposomes. Functionalized nanoliposomes targeted by specific binders can be a platform for further development of theranostics useful for imaging and solubilization of thrombi. Our next study is focused on labeling of binder-targeted liposomes with gadolinium complexes for in vivo imaging by MRI.

## Figures and Tables

**Table 1:** Amino acid sequences of selected protein binders. Sequence comparison of the fibrin binders. The non-mutated ABDwt was aligned with the randomized part of the ABD-derived binders selected by ribosome display. Grey boxes indicate the 11 positions at which the residues of ABD (aa 20–46) were randomized. The non-randomized N-terminal part of ABD (aa 1–19) contains sequence LAEAKVLANRELDKYGVSD.

Binder	20	21	22	23	24	25	26	27	28	29	30	31	32	33	34	35	36	37	38	39	40	41	42	43	44	45	46
**ABDwt**	**Y**	Y	K	N	**L**	I	N	**N**	A	**K**	**T**	V	**E**	**G**	V	K	**A**	**L**	I	D	**E**	I	L	A	A	L	P
**D7**	**A**	Y	K	N	**P**	I	N	**L**	A	**R**	**S**	V	**P**	**T**	V	K	**G**	**A**	I	D	**P**	I	L	A	A	L	P
**E7=F7**	**F**	Y	K	N	**L**	I	N	**V**	A	**M**	**P**	V	**V**	**L**	V	K	**T**	**A**	I	D	**G**	I	L	A	A	L	P
**F11**	**G**	Y	K	N	**W**	I	N	**P**	A	**D**	**G**	V	**A**	**G**	V	K	**S**	**A**	I	D	**A**	I	L	A	A	L	P

**Table 2:** Thermodynamic parameters of interaction between ABD-protein binders and metallochelation liposomes obtained by isothermal titration. Experimental conditions: 50 mM Tris buffer pH = 7.4; temperature of 25 °C (298.15 K).

Binder	K_d_ (M)	Δ°G (kJ/mol)
D7 F1	1.2 ± 0.1 × 10^−7^	−39.7 ± 0.7
ABD wt F2	1.06 ± 0.4 × 10^−7^	−39.8 ± 1.9
D7 F3	1.79 ± 0.55 × 10^−7^	−38.5 ± 2.8
D7 H2	2.21 ± 0.1 × 10^−9^	−49.4 ± 1.0
ABD-wt H2	2.35 ± 0.2 × 10^−8^	−43.5 ± 1.6

## Supplementary Materials

The following are available online at https://www.mdpi.com/1999-4923/11/12/642/s1, Figure S1: Binding of D7F1 binder to fibrin filaments of mouse thrombi, Figure S2: Binding of anti-insoluble fibrin antibodies to fibrin in the presence or absence of cysteine or to fibrinogen tested in ELISA, Figure S3: Binding of anti-fibrin monoclonal antibodies to BEP-carrying targets tested by ELISA, Figure S4: Binding of anti-fibrin monoclonal antibodies to differentially immobilized BEP targets tested by ELISA, Figure S5: Binding of mouse anti-insoluble fibrin monoclonal antibody to human fibrinogen and fibrin layers produced from a lyophilized product or from human plasma tested in ELISA, Figure S6: Thermal stability of D7-TolA-Avi protein.

## References

[1] Skaf E., Stein P.D., Beemath A., Sanchez J., Bustamante M.A., Olson R.E. (2005). Venous thromboembolism in patients with ischemic and hemorrhagic stroke. Am. J. Cardiol..

[2] Silvain J., Bellemain A., Ecollan P., Montalescot G., Collet J.P. (2011). Myocardial infarction: Role of new antiplatelet agents. Presse Med..

[3] Doherty S. (2017). Pulmonary embolism an update. Aust. Fam. Phys..

[4] Wendelboe A.M., Raskob G.E. (2016). Global burden of thrombosis: Epidemiologic aspects. Circ. Res..

[5] Heidt T., Ehrismann S., Hovener J.B., Neudorfer I., Hilgendorf I., Reisert M., Hagemeyer C.E., Zirlik A., Reinohl J., Bode C. (2016). Molecular imaging of activated platelets allows the detection of pulmonary embolism with magnetic resonance imaging. Sci. Rep..

[6] Von zur Muhlen C., Peter K., Ali Z.A., Schneider J.E., McAteer M.A., Neubauer S., Channon K.M., Bode C., Choudhury R.P. (2009). Visualization of activated platelets by targeted magnetic resonance imaging utilizing conformation-specific antibodies against glycoprotein iib/iiia. J. Vasc. Res..

[7] Zhang N., Li C., Zhou D., Ding C., Jin Y., Tian Q., Meng X., Pu K., Zhu Y. (2018). Cyclic rgd functionalized liposomes encapsulating urokinase for thrombolysis. Acta Biomater..

[8] Gargan P.E., Gaffney P.J., Pleasants J.R., Ploplis V.A. (1993). A monoclonal antibody which recognises an epitopic region unique to the intact fibrin polymeric structure. Fibrinolysis.

[9] Soe G., Kohno I., Inuzuka K., Itoh Y., Matsuda M. (1996). A monoclonal antibody that recognizes a neo-antigen exposed in the e domain of fibrin monomer complexed with fibrinogen or its derivatives: Its application to the measurement of soluble fibrin in plasma. Blood.

[10] Wada H., Kobayashi T., Abe Y., Hatada T., Yamada N., Sudo A., Uchida A., Nobori T. (2006). Elevated levels of soluble fibrin or d-dimer indicate high risk of thrombosis. J. Thromb. Haemost..

[11] Doh H.J., Song K.S., Kang M.S., Kim D.S., Kim K.A., Kang J., Jang Y., Chung K.H. (2006). Novel monoclonal antibody that recognizes new neoantigenic determinant of d-dimer. Thromb. Res..

[12] Kolodziej A.F., Nair S.A., Graham P., McMurry T.J., Ladner R.C., Wescott C., Sexton D.J., Caravan P. (2012). Fibrin specific peptides derived by phage display: Characterization of peptides and conjugates for imaging. Bioconjugate Chem..

[13] Overoye-Chan K., Koerner S., Looby R.J., Kolodziej A.F., Zech S.G., Deng Q., Chasse J.M., McMurry T.J., Caravan P. (2008). Ep-2104r: A fibrin-specific gadolinium-based mri contrast agent for detection of thrombus. J. Am. Chem. Soc..

[14] Vymazal J., Spuentrup E., Cardenas-Molina G., Wiethoff A.J., Hartmann M.G., Caravan P., Parsons E.C. (2009). Thrombus imaging with fibrin-specific gadolinium-based mr contrast agent ep-2104r: Results of a phase ii clinical study of feasibility. Investig. Radiol..

[15] Pilch J., Brown D.M., Komatsu M., Jarvinen T.A., Yang M., Peters D., Hoffman R.M., Ruoslahti E. (2006). Peptides selected for binding to clotted plasma accumulate in tumor stroma and wounds. Proc. Natl. Acad. Sci. USA.

[16] Hisada Y., Yasunaga M., Hanaoka S., Saijou S., Sugino T., Tsuji A., Saga T., Tsumoto K., Manabe S., Kuroda J. (2013). Discovery of an uncovered region in fibrin clots and its clinical significance. Sci. Rep..

[17] Fuchigami H., Manabe S., Yasunaga M., Matsumura Y. (2018). Chemotherapy payload of anti-insoluble fibrin antibody-drug conjugate is released specifically upon binding to fibrin. Sci. Rep..

[18] Obonai T., Fuchigami H., Furuya F., Kozuka N., Yasunaga M., Matsumura Y. (2016). Tumour imaging by the detection of fibrin clots in tumour stroma using an anti-fibrin fab fragment. Sci. Rep..

[19] Tiukinhoy-Laing S.D., Buchanan K., Parikh D., Huang S., MacDonald R.C., McPherson D.D., Klegerman M.E. (2007). Fibrin targeting of tissue plasminogen activator-loaded echogenic liposomes. J. Drug Target..

[20] Klegerman M.E., Zou Y., McPherson D.D. (2008). Fibrin targeting of echogenic liposomes with inactivated tissue plasminogen activator. J. Liposome Res..

[21] Yan J.P., Ko J.H., Qi Y.P. (2004). Generation and characterization of a novel single-chain antibody fragment specific against human fibrin clots from phage display antibody library. Thromb. Res..

[22] Putelli A., Kiefer J.D., Zadory M., Matasci M., Neri D. (2014). A fibrin-specific monoclonal antibody from a designed phage display library inhibits clot formation and localizes to tumors in vivo. J. Mol. Biol..

[23] Koudelka S., Mikulik R., Masek J., Raska M., Turanek Knotigova P., Miller A.D., Turanek J. (2016). Liposomal nanocarriers for plasminogen activators. J. Control. Release.

[24] Nisini R., Poerio N., Mariotti S., De Santis F., Fraziano M. (2018). The multirole of liposomes in therapy and prevention of infectious diseases. Front. Immunol..

[25] Belfiore L., Saunders D.N., Ranson M., Thurecht K.J., Storm G., Vine K.L. (2018). Towards clinical translation of ligand-functionalized liposomes in targeted cancer therapy: Challenges and opportunities. J. Control. Release.

[26] Kumar R., Dogra S., Amarji B., Singh B., Kumar S., Sharma S., Vinay K., Mahajan R., Katare O.P. (2016). Efficacy of novel topical liposomal formulation of cyclosporine in mild to moderate stable plaque psoriasis: A randomized clinical trial. JAMA Dermatol..

[27] Bartheldyova E., Turanek Knotigova P., Zachova K., Masek J., Kulich P., Effenberg R., Zyka D., Hubatka F., Kotoucek J., Celechovska H. (2019). N-oxy lipid-based click chemistry for orthogonal coupling of mannan onto nanoliposomes prepared by microfluidic mixing: Synthesis of lipids, characterisation of mannan-coated nanoliposomes and in vitro stimulation of dendritic cells. Carbohydr. Polym..

[28] Ramasamy T., Ruttala H.B., Gupta B., Poudel B.K., Choi H.-G., Yong C.S., Kim J.O. (2017). Smart chemistry-based nanosized drug delivery systems for systemic applications: A comprehensive review. J. Control. Release.

[29] Bartheldyova E., Effenberg R., Masek J., Prochazka L., Knotigova P.T., Kulich P., Hubatka F., Velinska K., Zelnickova J., Zouharova D. (2018). Hyaluronic acid surface modified liposomes prepared via orthogonal aminoxy coupling: Synthesis of nontoxic aminoxylipids based on symmetrically alpha-branched fatty acids, preparation of liposomes by microfluidic mixing, and targeting to cancer cells expressing cd44. Bioconjugate Chem..

[30] Ahmad J.N., Li J., Biedermannova L., Kuchar M., Sipova H., Semeradtova A., Cerny J., Petrokova H., Mikulecky P., Polinek J. (2012). Novel high-affinity binders of human interferon gamma derived from albumin-binding domain of protein g. Proteins.

[31] Mareckova L., Petrokova H., Osicka R., Kuchar M., Maly P. (2015). Novel binders derived from an albumin-binding domain scaffold targeting human prostate secretory protein 94 (psp94). Protein Cell.

[32] Hlavnickova M., Kuchar M., Osicka R., Vankova L., Petrokova H., Maly M., Cerny J., Arenberger P., Maly P. (2018). Abd-derived protein blockers of human il-17 receptor a as non-igg alternatives for modulation of il-17-dependent pro-inflammatory axis. Int. J. Mol. Sci..

[33] Kuchar M., Vankova L., Petrokova H., Cerny J., Osicka R., Pelak O., Sipova H., Schneider B., Homola J., Sebo P. (2014). Human interleukin-23 receptor antagonists derived from an albumin-binding domain scaffold inhibit il-23-dependent ex vivo expansion of il-17-producing t-cells. Proteins.

[34] Krizova L., Kuchar M., Petrokova H., Osicka R., Hlavnickova M., Pelak O., Cerny J., Kalina T., Maly P. (2017). P19-targeted abd-derived protein variants inhibit il-23 binding and exert suppressive control over il-23-stimulated expansion of primary human il-17+ t-cells. Autoimmunity.

[35] Mašek J., Bartheldyová E., Turánek-Knotigová P., Škrabalová M., Korvasová Z., Plocková J., Koudelka Š., Škodová P., Kulich P., Křupka M. (2011). Metallochelating liposomes with associated lipophilised norabumdp as biocompatible platform for construction of vaccines with recombinant his-tagged antigens: Preparation, structural study and immune response towards rhsp90. J. Control. Release.

[36] Křupka M., Mašek J., Bartheldyová E., Knötigová P.T., Plocková J., Korvasová Z., Škrabalová M., Koudelka Š., Kulich P., Zachová K. (2012). Enhancement of immune response towards non-lipidized borrelia burgdorferi recombinant ospc antigen by binding onto the surface of metallochelating nanoliposomes with entrapped lipophilic derivatives of norabumdp. J. Control. Release.

[37] Zuev Y.F., Litvinov R.I., Sitnitsky A.E., Idiyatullin B.Z., Bakirova D.R., Galanakis D.K., Zhmurov A., Barsegov V., Weisel J.W. (2017). Conformational flexibility and self-association of fibrinogen in concentrated solutions. J. Phys. Chem. B.

[38] Koo J., Galanakis D., Liu Y., Ramek A., Fields A., Ba X., Simon M., Rafailovich M.H. (2012). Control of anti-thrombogenic properties: Surface-induced self-assembly of fibrinogen fibers. Biomacromolecules.

[39] Lounes K.C., Lefkowitz J.B., Henschen-Edman A.H., Coates A.I., Hantgan R.R., Lord S.T. (2001). The impaired polymerization of fibrinogen longmont (bbeta166arg-->cys) is not improved by removal of disulfide-linked dimers from a mixture of dimers and cysteine-linked monomers. Blood.

[40] Schwartz M.L., Pizzo S.V., Hill R.L., McKee P.A. (1971). The effect of fibrin-stabilizing factor on the subunit structure of human fibrin. J. Clin. Investig..

[41] Zadravec P., Mareckova L., Petrokova H., Hodnik V., Perisic Nanut M., Anderluh G., Strukelj B., Maly P., Berlec A. (2016). Development of recombinant lactococcus lactis displaying albumin-binding domain variants against shiga toxin 1 b subunit. PLoS ONE.

[42] Wahl V., Saurugger E., Khinast J., Laggner P. (2015). Specific surface, crystallinity, and dissolution of lyophilized fibrinogen. A study by combined small- and wide-angle x-ray scattering (swaxs). Eur. J. Pharm. Biopharm..

[43] Wahl V., Leitgeb S., Laggner P., Pichler H., Liebminger A., Khinast J. (2015). The influence of residual water on the solid-state properties of freeze-dried fibrinogen. Eur. J. Pharm. Biopharm..

[44] Křupka M., Mašek J., Barkocziová L., Knotigová P.T., Kulich P., Plockova J., Lukac R., Bartheldyová E., Koudelka S., Chaloupková R. (2016). The position of his-tag in recombinant ospc and application of various adjuvants affects the intensity and quality of specific antibody response after immunization of experimental mice. PLoS ONE.

[45] Mašek J., Bartheldyová E., Korvasová Z., Škrabalová M., Koudelka Š., Kulich P., Kratochvílová I., Miller A.D., Ledvina M., Raška M. (2011). Immobilization of histidine-tagged proteins on monodisperse metallochelation liposomes: Preparation and study of their structure. Anal. Biochem..

[46] Platt V., Huang Z., Cao L., Tiffany M., Riviere K., Szoka F.C. (2010). Influence of multivalent nitrilotriacetic acid lipid-ligand affinity on the circulation half-life in mice of a liposome-attached his6-protein. Bioconjugate Chem..

