# Supplementary material for: Targeting Human Thrombus by Liposomes Modified with Anti-Fibrin Protein Binders
Source: Pharmaceutics. 2019 Dec 2;11(12):642. doi: 10.3390/pharmaceutics11120642 (PMC6955937; doi:10.3390/pharmaceutics11120642)
Supplement: Supplementary file 1 [file pharmaceutics-11-00642-s001.pdf]

pharmaceutics

MDPI

Supplementary Materials: Targeting Human Thrombus by Liposomes Modified with Anti-Fibrin Protein Binders

Hana Petroková, Josef Mašek, Milan Kuchař, Andrea Vítečková Wünschová, Jana Štikarová, Eliška Bartheldyová, Pavel Kulich, František Hubatka, Jan Kotouček, Pavlína Turánek Knotigová, Eva Vohlídalová, Renata Héžová, Eliška Mašková, Stuart Macaulay, Jan Evangelista Dyr, Milan Raška, Robert Mikulík, Petr Malý and Jaroslav Turánek

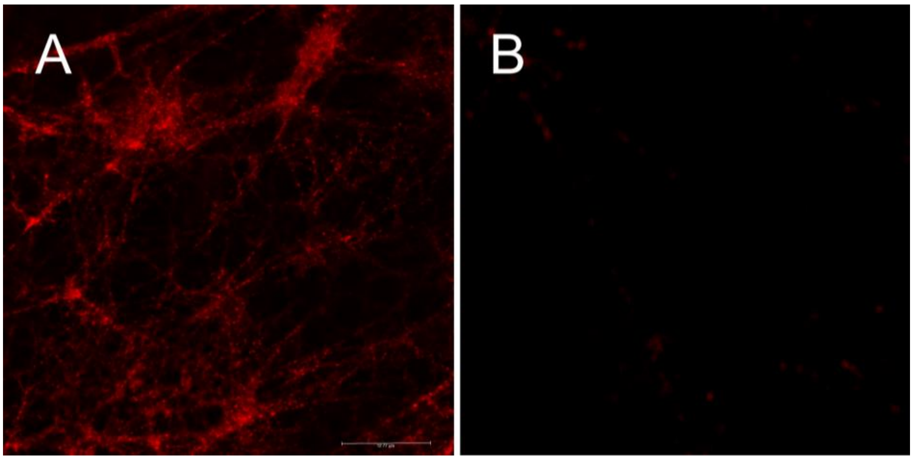

Figure S1. Binding of D7F1 binder to fibrin filaments of mouse thrombi. D7F1 protein binder was incubated with mouse thrombus, washed and visualised using fluorescently labelled ANTI-FLAG® antibody (**A**). ABDwt visualised using fluorescently labelled ANTI-FLAG® antibody was used as a negative control (**B**).

Methods: Mouse thrombi were prepared using the same protocol as described in the main text of the publication.

Preparation of thrombi from whole mouse blood. Thrombi were prepared from 10 μL of whole mouse blood without addition of anticoagulants in glass tubes – Target Insert 300 μL Capacity (National Scientific Supply Company Inc., Claremont, CA, USA) at room temperature for 4 hours. Before a start of an experiment each thrombus was gently washed with 2 ml of TBS buffer.

Confocal microscopy of D7F1 targeted fibrin filaments. D7F1 (final concentration of 20 μg/mL) was incubated with prepared thrombus in TBS buffer for 30 min. After that, each thrombus was washed twice by 200 μL of TBS buffer and placed into TBS buffer with monoclonal ANTI-FLAG® M-Cy3™, Clone M2 (Sigma-Aldrich), respectively. The concentration was 10 μg/mL and the incubation lasted one hour.

Conclusion: The binding of D7F1 protein binder was observed in mouse thrombi, however the detected signal was significantly lower as compared to human thrombi.

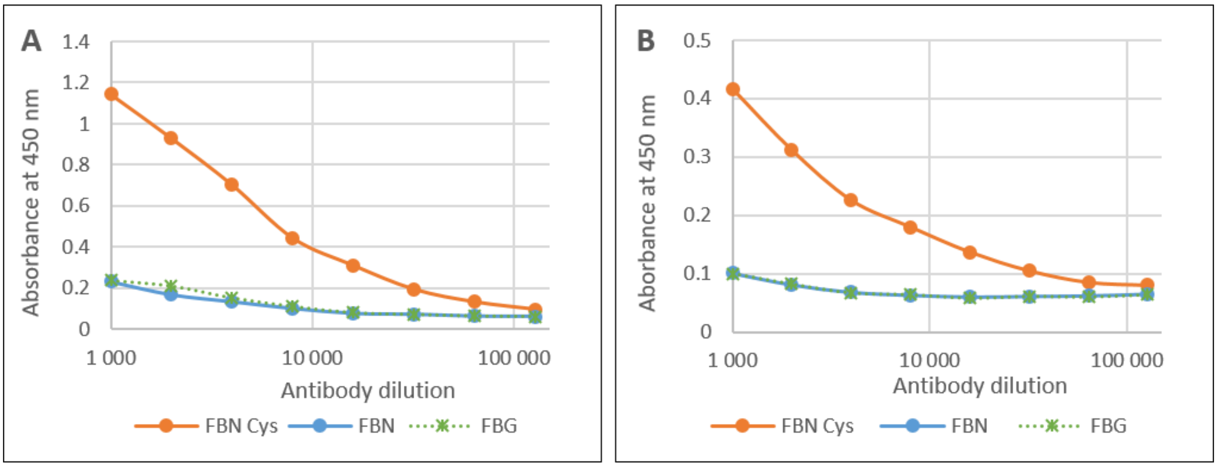

Figure S2. Binding of anti-insoluble fibrin antibodies to fibrin in the presence or absence of cysteine or to fibrinogen tested in ELISA. Fibrin was prepared by the cleavage of fibrinogen by thrombin in the presence or absence of cystein. (A) Binding of anti-insoluble fibrin rat monoclonal antibody (clone 443) and (B) mouse monoclonal antibody (clone L, 1101) to coated fibrin (FBN), fibrin formed in the presence of cysteine (FBN Cys) or to soluble fibrinogen (FBG) was monitored by secondary anti-rat-IgG-HRP and anti-mouse-IgG-HRP, respectively. TMB was used as a substrate for visualization by HRP.

Conclusion: Both monoclonal antibodies significantly bind to fibrin prepared in the presence of cysteine and do not substantially bind to fibrinogen nor fibrin formed in the absence of cysteine.

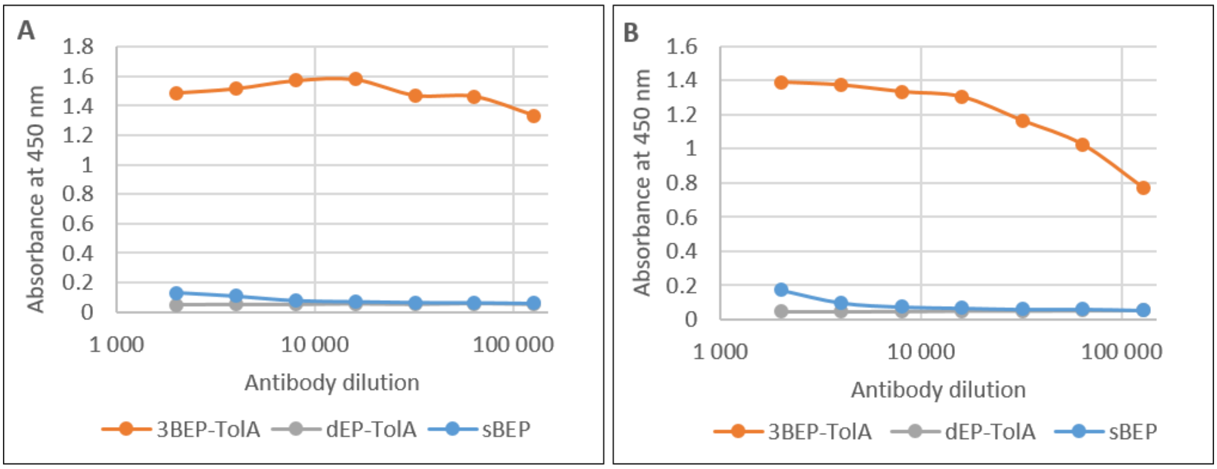

Figure S3. Binding of anti-fibrin monoclonal antibodies to BEP-carrying targets tested by ELISA. Recombinant BEP fusion protein (3BEP-TolA-Avi), 16 amino acid synthetic BEP peptide (sBEP) or a control fusion protein lacking BEP sequences (ΔEP-TolA-Avi) were used to coat wells of 96-well microtitre plate. Binding of anti-insoluble fibrin rat monoclonal antibody (clone 443) (panel **A**) or mouse monoclonal antibody (clone L, 1101) (panel **B**) to coated targets was monitored by secondary anti-rat-IgG-HRP and anti-mouse-IgG-HRP, respectively. TMB was used as a substrate for visualization by HRP.

Conclusion: Both monoclonal antibodies bind to the recombinant protein 3BEP-TolA while no binding to protein lacking BEP epitope(ΔEP-TolA) was detected, thus confirming their specificity. Absence of binding to sBEP suggests that the peptide may not be in a proper conformation required for the antibody recognition.

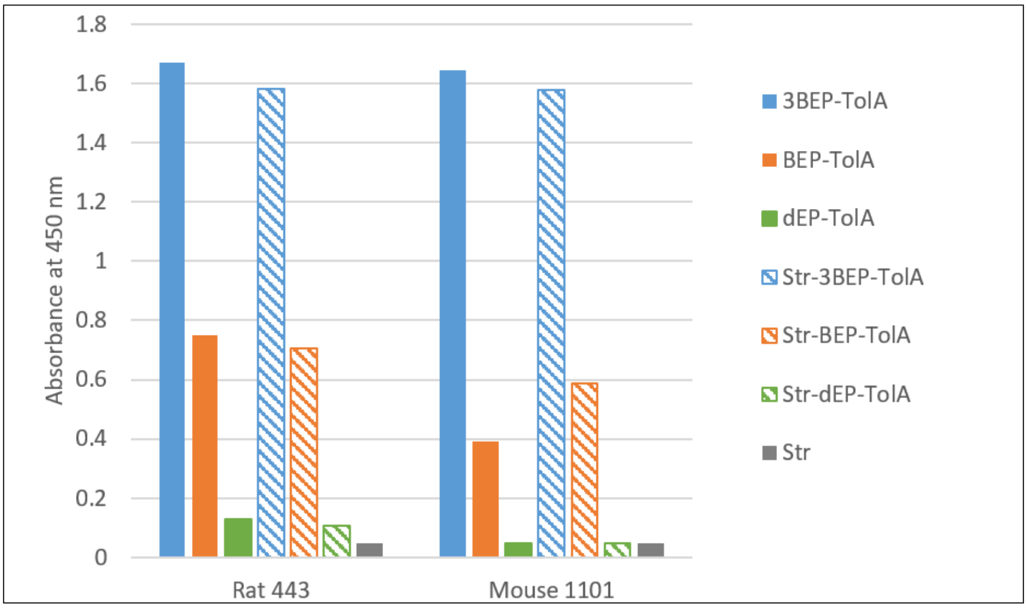

Figure S4. Binding of anti-fibrin monoclonal antibodies to differentially immobilized BEP targets tested by ELISA. In vivo biotinylated 3BEP-TolA-Avi, BEP-TolA-Avi and ΔEP-TolA-Avi recombinant proteins were used for a direct coating of wells in 96-well microplate or for a C-terminally-oriented immobilization via bound streptavidine (Str-3BEP-TolA-Avi, Str-BEP-TolA-Avi, Str-ΔEP-TolA-Avi). Binding of anti-insoluble fibrin rat monoclonal antibody (clone 443) or mouse monoclonal antibody (clone L, 1101) to immobilized BEP targets was monitored by secondary anti-rat-IgG-HRP and anti-mouse-IgG-HRP, respectively. Binding to a coated streptavidine (Str) as a negative control was also monitored. TMB was used as a substrate for visualization by HRP.

Conclusion: Different set up for the immobilization of BEP protein targets has no effect on binding function as both layouts provide the similar binding effect. mAbs bind to 3BEP-TolA more than to BEP-TolA thus confirming the specificity of the Bβ epitope recognition.

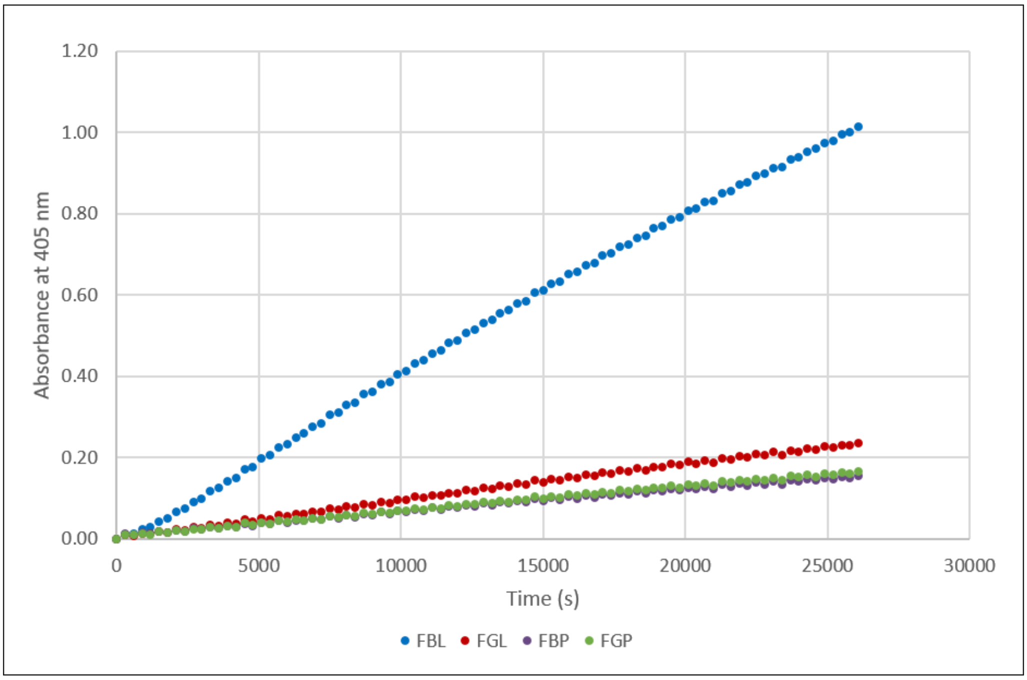

Figure S5. Binding of mouse anti-insoluble fibrin monoclonal antibody to human fibrinogen and fibrin layers produced from a lyofilized product or from human plasma tested in ELISA. Binding of mouse monoclonal antibody clone L (1101) to fibrinogen (FGL), human plasma (FGP), fibrin layer from lyophilized fibrinogen (FBL) and to that prepared from a human plasma (FBP). For detection,

anti-mouse IgG conjugated with alkaline phosphatase and para-nitrophenylphosphate as a substrate was used. Absorbance was monitored at 405 nm.

Conclusion: Significant binding of mouse monoclonal antibody was found only for the fibrin layer prepared from a lyophilized fibrinogen product.

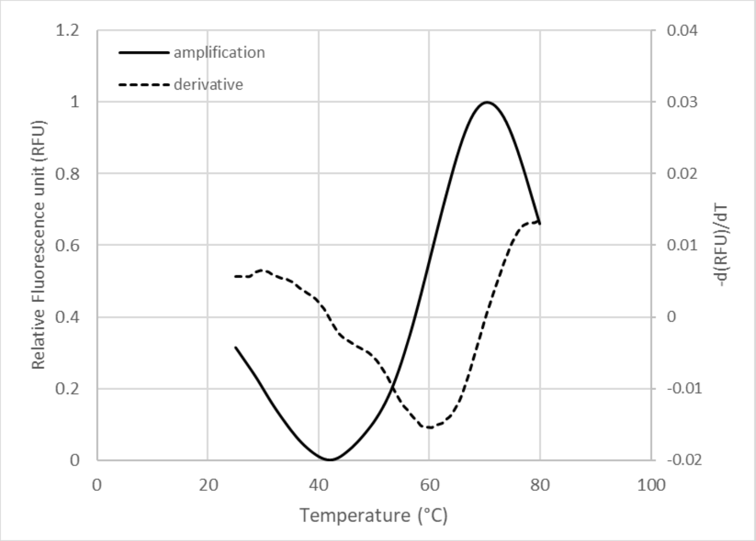

Figure S6. Thermal stability of D7-TolA-Avi protein. The stability of the protein was measured by thermal shift assay using SyproOrange dye. Melting temperature was determined as a minimum of the first derivative curve (dashed line), found to be 60 °C.

Conclusion: To investigate thermal stability of the D7 protein variant, thermal shift assay (TSA) using SyproOrange fluorescent dye was performed. Calculated melting temperature was found to be 60 °C (Figure S5). In our previous studies [29] we provided a temperature melting point for the parental non-mutated ABD-TolA-Avi wild–type protein (58 °C). Thus, randomization of 11 amino acid residues of the ABD scaffold in the case of the D7 protein variant did not result in a loss of the general protein stability.
